# The impact of age-related dysregulation of the angiotensin system on mitochondrial redox balance

**DOI:** 10.3389/fphys.2014.00439

**Published:** 2014-11-24

**Authors:** Ramya Vajapey, David Rini, Jeremy Walston, Peter Abadir

**Affiliations:** ^1^School of Medicine, Northeast Ohio Medical UniversityRootstown, OH, USA; ^2^Division of Cellular and Molecular Medicine, Art as Applied to Medicine, Johns Hopkins UniversityBaltimore, MD, USA; ^3^Division of Geriatrics Medicine and Gerontology, Department of Medicine, Johns Hopkins UniversityBaltimore, MD, USA

**Keywords:** mitochondria, angiotensin II, angiotensin II type 1 receptor blockers, mitochondrial angiotensin system, redox regulation, aging, frailty

## Abstract

Aging is associated with the accumulation of various deleterious changes in cells. According to the free radical and mitochondrial theory of aging, mitochondria initiate most of the deleterious changes in aging and govern life span. The failure of mitochondrial reduction-oxidation (redox) homeostasis and the formation of excessive free radicals are tightly linked to dysregulation in the Renin Angiotensin System (RAS). A main rate-controlling step in RAS is renin, an enzyme that hydrolyzes angiotensinogen to generate angiotensin I. Angiotensin I is further converted to Angiotensin II (Ang II) by angiotensin-converting enzyme (ACE). Ang II binds with equal affinity to two main angiotensin receptors—type 1 (AT_1_R) and type 2 (AT_2_R). The binding of Ang II to AT_1_R activates NADPH oxidase, which leads to increased generation of cytoplasmic reactive oxygen species (ROS). This Ang II-AT_1_R–NADPH-ROS signal triggers the opening of mitochondrial K_ATP_ channels and mitochondrial ROS production in a positive feedback loop. Furthermore, RAS has been implicated in the decrease of many of ROS scavenging enzymes, thereby leading to detrimental levels of free radicals in the cell. AT_2_R is less understood, but evidence supports an anti-oxidative and mitochondria-protective function for AT_2_R. The overlap between age related changes in RAS and mitochondria, and the consequences of this overlap on age-related diseases are quite complex. RAS dysregulation has been implicated in many pathological conditions due to its contribution to mitochondrial dysfunction. Decreased age-related, renal and cardiac mitochondrial dysfunction was seen in patients treated with angiotensin receptor blockers. The aim of this review is to: (a) report the most recent information elucidating the role of RAS in mitochondrial redox hemostasis and (b) discuss the effect of age-related activation of RAS on generation of free radicals.

## Mitochondria and angiotensin system: overview

It is well accepted that mitochondria are the major source of ATP, which is the fuel for many cellular processes. However, mitochondrial function extends well beyond bioenergetics (Kang and Pervaiz, 2012). Accumulating evidence suggests that mitochondria are also signaling organelles that interact with the rest of the cell through ROS and an array of other signaling complexes (Shigenaga et al., 1994; Turrens, 2003; Chandel, 2014). Earlier studies suggested that mitochondrial ROS were detrimental byproducts associated with several pathological conditions (Turrens, 2003; Chandel, 2014). However, more recent studies suggest that ROS may have fundamental cellular functions—acting as signaling molecules (Chandel, 2014), stabilizing hypoxia inducible factors (HIFs), and inducing gene expression to promote cellular adaptation to low oxygen levels (Chandel et al., 1998, 2000a). ROS also play a role in tumor necrosis factor (TNF) receptor signaling (Chandel et al., 2000b, 2001; Nemoto et al., 2000; Chandel, 2014) and defense against pathogens (Yu et al., 2012).

RAS is a key hormonal pathway that affects virtually every organ. While many of the endocrine (circulating), paracrine (cell-to-different cell), and autocrine (cell-to-same cell) effects of the RAS are believed to be mediated through the canonical extracellular RAS, an independent and differentially regulated intracellular RAS has also been proposed (Robertson and Khairallah, 1971; Inagami et al., 1986, 1988, 1990; Hunt et al., 1992; Sadoshima et al., 1993; Mercure et al., 1998; Vila-Porcile and Corvol, 1998; Cook et al., 2001; Sherrod et al., 2005; Kumar et al., 2007, 2008, 2012; Peters, 2008, 2012; Abadir et al., 2011, 2012). The major components of RAS are (1) angiotensinogen, derived from the liver; (2) renin, derived from the juxtaglomerular cells of afferent arterioles; (3) angiotensin converting enzyme (ACE), a dipeptidyl carboxypeptidase; (4) angiotensin II, and (5) angiotensin II receptors. Angiotensinogen is a globular protein that serves as a substrate for renin, a glycoproteolytic enzyme. The first step of the RAS pathway is conversion of angiotensinogen to angiotensin I by renin. Angiotensin I is then converted to angiotensin II via ACE enzyme (Griendling et al., 1993). At all levels -endocrine, paracrine, and autocrine—Ang II binds to two main receptor subtypes, AT_1_R and AT_2_R. Both belong to the G-protein-coupled receptor family but differ in terms of tissue distribution and cell signaling pathways. Ang II binds with equal affinity to AT_1_R and AT_2_R. These receptors, in turn, activate multiple signal transduction pathways that include signaling molecules like Nitric Oxide (NO^·^), Protein Tyrosine Phosphatases (PTP), and Mitogen-activated protein kinase (MAPK) (Abadir, 2011; Abadir et al., 2012). AT_1_R and AT_2_R stimulation generally leads to opposing actions (Abadir, 2011; Abadir et al., 2012) summarized in Table 1.

**Table 1:** **Opposing functions of AT1R and AT2R**.

**AT1R**	**AT2R**
Vasoconstriction	Vasodilation
↑ cell growth	↓ cell growth
Cellular proliferation	Cellular differentiation
Anti-naturetic	Naturetic
Production of O_2_	Production of NO
↑ fibroblast proliferation/collagen synthesis	↓ fibroblast proliferation
Pro-apoptotic	Anti-apoptotic

Evidence supporting an important role for RAS in mitochondrial function/dysfunction comes from many sources (Cook and Re, 2012; Ellis et al., 2012; Garcia et al., 2012; Gwathmey et al., 2012; Li et al., 2012; Singh et al., 2012; Wangler et al., 2012; Yu et al., 2012; Zaobornyj and Ghafourifar, 2012; Ferder et al., 2013; Sovari et al., 2013). The main function of mitochondria is to generate ATP via the electron transport chain. Electrons are transferred through complex I to complex IV with oxygen as the final electron acceptor. Damaged ETC complexes may no longer accept electrons, leading to generation of ROS (Kregel and Zhang, 2007). AT_1_R knock-out mice exhibit a notable phenotype with increased mitochondrial numbers and average lifespan extension exceeding 25% (Benigni et al., 2009). Other clues of the influence of RAS on mitochondria may be gleaned from prior work, which demonstrated that Ang II infusion in rodents induced cardiomyopathy by increasing mitochondrial ROS generation. In these animals, overexpression of catalase specific for mitochondria, but not peroxisomes, protected them against cardiac hypertrophy, fibrosis, and diastolic dysfunction (Dai et al., 2009; Dikalov and Nazarewicz, 2013). The recent identification of a functional intra-mitochondrial angiotensin system (MAS) provided additional insight into the RAS interface with mitochondria (Eto et al., 2002; Kumar et al., 2008; Abadir et al., 2011, 2012). Recently, changes in MAS and a novel role for AT_1_R on mitochondrial respiration in diabetes were reported (Persson et al., in press). In mitochondria from renal tubular cells, expression of AT_2_R decreases while AT_1_R increases with age. Chronic administration of losartan, an angiotensin receptor blocker, prevented age-related decrease of mitochondrial AT_2_R. Other parts of the system including renin and ACE have also been localized intracellularly, with evidence suggesting their presence in the nucleus and mitochondria (Vidotti et al., 2004; Abadir et al., 2012). Interestingly, this intracellular system is independent of circulating RAS; ACE inhibitors fail to block intracellular ACE (Cristovam et al., 2008).

Several groups demonstrated a tight link between RAS, mitochondria, and a host of age-related pathologic conditions (Inagami, 2011; Carey, 2012; Conti et al., 2012; Cook and Re, 2012; Dai et al., 2012; Ellis et al., 2012; Gao et al., 2012; Garcia et al., 2012; Gwathmey et al., 2012; Horan et al., 2012; Li et al., 2012; Singh et al., 2012; Wangler et al., 2012; Yu et al., 2012; Zaobornyj and Ghafourifar, 2012). In addition, RAS dysregulation aggravates several acute and chronic diseases, many of which have been linked to mitochondrial dysfunction [atherosclerosis (Warnholtz et al., 1999), kidney disease (Ma et al., 1998), myocardial damage after infarction (Kuno et al., 2002), cerebral infarct size after ischemia (Panahpour and Dehghani, 2012)]. Locally activated RAS in heart tissue has been implicated in cardiac hypertrophy and fibrosis (Kumar et al., 2008). The mechanism by which intracellular angiotensin II (iAng II) affects cardiac tissue has been a topic of debate for years. One possible mechanism is that iAng II interacts with intracellular AT_1_R or AT_1_-like receptors to bring about the observed changes. Another mechanism involves iAng II binding directly to chromatin to promote the transcription of growth factors like insulin, PDGF, and FGF-2 (Baker et al., 2004). iAng II may also may also affect Ca^+2^ fluxes and activate phospholipase C and PKC by binding to AT_1_R on sarcolemma, mitochondria, or internalized receptors (Eto et al., 2002; Baker et al., 2004).

Similar to extracellular RAS, intracellular RAS has been implicated in many pathological conditions as well. Intracellularly, RAS is highly active in producing increased amounts of iAng II in mice with advanced heart failure. Increased ventricular hypertrophy or fibrosis was also observed (De Mello and Gerena, 2008). The mechanism by which Ang II is produced has not been defined yet. Pressure overload and mechanical stretch of cardiomyocytes after myocardial infarction may cause secretion of local Ang II. After examination of levels of expression of angiotensinogen, renin, ACE and AT_1_ genes, stretched cardiac myocytes were observed to have higher levels of mRNA and RAS enzymes than un-stretched myocytes (Malhotra et al., 1999). This increased Ang II seems to work through a mineralocorticoid receptor because the administration of an aldosterone receptor antagonist mitigated the effect of Ang II on inward Ca^+2^ current in failing hearts (De Mello and Gerena, 2008). Ang II further caused cell swelling in failing cardiac myocytes via activation of ionic channels and decrease in gap junction permeability. These molecular changes may lead to altered gene expression, which could be contributing to cardiac remodeling. In addition, changes in ionic and gap junction permeability can decrease action potential duration and conduction velocity. All of these factors may lead to cardiac arrhythmias by causing electrical uncoupling, mechanical de-synchronization, and cardiac remodeling (De Mello and Frohlich, 2011). Furthermore, diabetic patients seem to have upregulated intracellular RAS activity because high glucose in rat mesangial cells resulted in ~30-fold increase in intracellular renin (iRenin) activity and increased iAng II concentrations localized mostly to the nucleus. Localization of iAng II to the nucleus suggests that the mechanism of action of iAng II is both cytoplasmic and nuclear. Renin and chymase (an alternative Ang II-generating enzyme), were implicated in the glucose-induced increase in Ang II rather than ACE; this confirms that the increased Ang II is not due to increased uptake of circulating Ang II but rather due to localized tissue synthesis (Re et al., 1984; Vidotti et al., 2004; Kumar et al., 2007).

## Mitochondrial reduction-oxidation (redox) balance: role of RAS in ROS generation, transport and elimination

Mitochondria play a critical role in redox chemistry. Mitochondrial redox balance is the process by which, under physiological conditions, mitochondria maintain a dynamic balance between ROS generation, their transport, and an array of antioxidant systems (glutathione, glutathione peroxidase, glutathione reductase, MnSOD, catalase, and thioredoxin system) in response to fluctuations in cellular energy demand (Aon et al., 2010; Cortassa et al., 2014).

### ROS generation and transport

ROS are generated from various sources including NADPH oxidase (NOX2 and NOX4), uncoupled nitric oxide synthase (NOS), xanthine oxidase (XO), and mitochondria. Of these, mitochondria are the main source of ROS (Nickel et al., 2014).

ROS are generated in the respiratory chain, mainly at the level of complex I and III (Murphy, 2009; Kembro et al., 2014) although recent evidence also involves complex II (Drose, 2013).

Electron transfer between the respiratory complexes in the respiratory chain generates a proton motive force composed of proton and electrical gradients that then drives ATP synthesis at the level of ATP synthase. NADH and FADH_2_, generated in the Tricarboxylic Acid (TCA) cycle act as electron donors for the electron transport chain (ETC) (Rich and Marechal, 2010). Oxygen is the final acceptor of four electrons transferred from the ETC, and converted to H_2_O (Mitchell, 1961; Liu et al., 2002). If less than four electrons are transferred to oxygen, ROS are produced (Kregel and Zhang, 2007). Damaged ETC complexes may no longer accept electrons. Excess ROS can damage respiratory complexes and initiate a vicious cycle of ROS overflow thus highlighting the fact that mitochondria can be both source and victim of oxidative stress (Daiber, 2010).

ADP and Ca^+2^, can modulate the rate of ATP generation according to energy demand. ADP stimulates ATP synthesis via F1F0 ATPase, driven mainly by the electrical component of the proton motive force (Wood, 2006). The dissipation of the mitochondrial membrane potential generates a “pull” of electrons from NADH at the level of complex I, or FADH2 from succinate at the level of complex II thus increasing O_2_ consumption.

As a matter of fact, an increase in energy demand, e.g., higher cardiac workload under exercising conditions, will increase both ADP and Ca^2+^ uptake by the mitochondria to increase ATP supply to match the demand (Cortassa et al., 2006; Murphy, 2009). Energized mitochondria will exhibit higher levels of ATP and NAD(P)H and lower electron flow thus increasing the probability of O^·−^_2_generation in the respiratory chain. Consequently, mitochondrial ROS production is highly dependent on the energetic and redox status of mitochondria (Kang and Pervaiz, 2012; Cortassa et al., 2014).

### RAS-induced mitochondrial ROS generation

**Figure 1:**
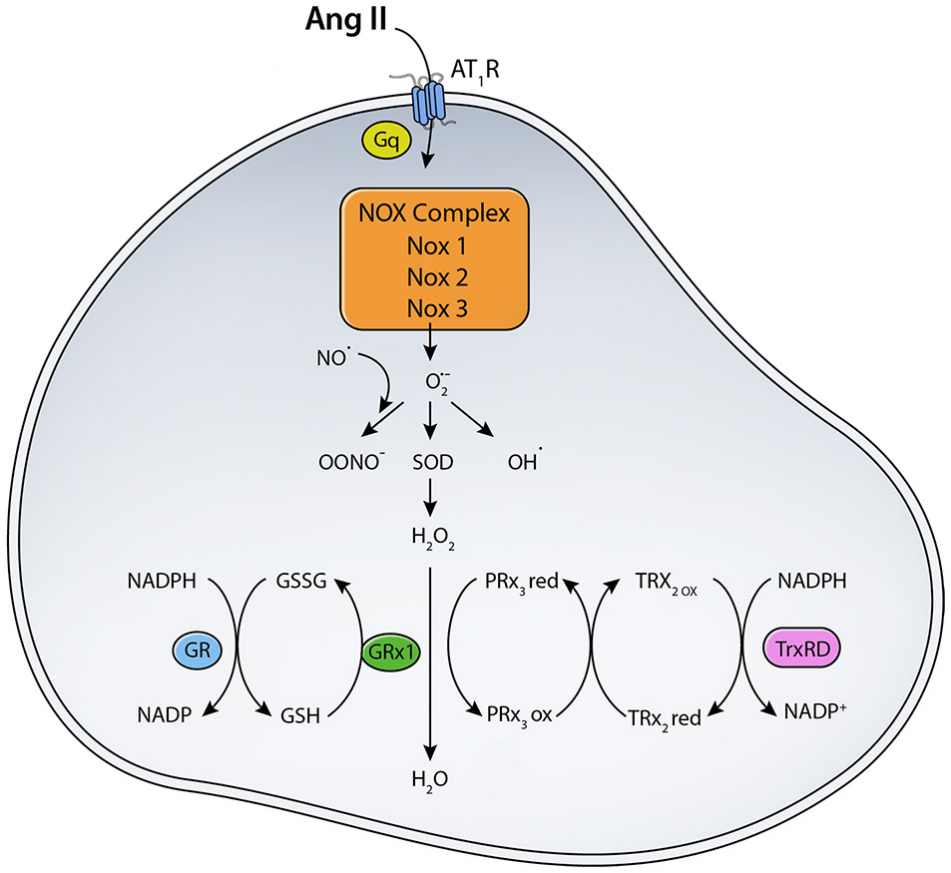
**Scheme for RAS induced ROS generation**. The binding of Ang II to AT_1_R activates NADPH oxidase that transfers an electron from NADPH to O_2_ generating O^·−^_2_. Location and expression level of different NOX enzymes determine their function. (SOD, Superoxide dismutase; TrxRD, thioredoxin reductase; GR, Glutathione Reductase; GPX, Glutathione peroxidase; PRx3, peroxiredoxin 3; TRx2, thioredoxin).

**Figure 2:**
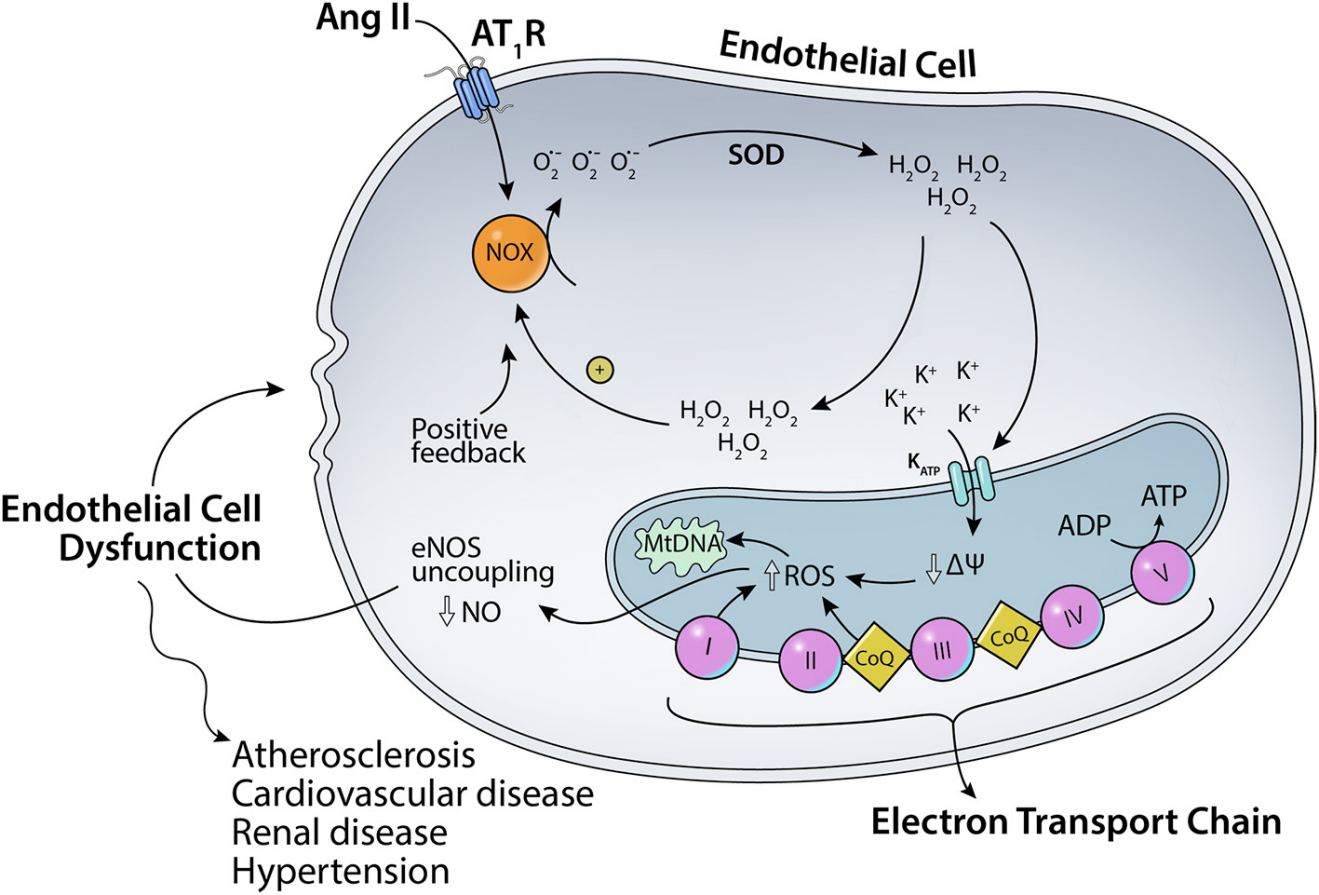
**Scheme for the effects of Ang II on mitochondrial K_ATP_ channels**. Ang II-AT_1_R–NADPH-ROS signal triggers the opening of mitochondrial K_ATP_ channels and mitochondrial ROS production in a positive feedback loop.

As mentioned before, angiotensin II can bind to two major receptors: AT_1_R and AT_2_R. Ang II binding to AT_1_R in the plasma membrane has been implicated in increased ROS production (Figure 1). Ang II-AT_1_R can activate NADPH oxidase, leading to increased generation of cytoplasmic ROS. This Ang II-AT_1_R–NADPH-ROS signal triggers the opening of mitochondrial K_ATP_ (mtK_ATP_) channels (Figure 2) that in turn activates mitochondrial ROS production in a positive feedback loop (Daiber, 2010). Opening of mtK_ATP_ channels decreases mitochondrial membrane potential. This triggers the opening of mitochondrial permeability transition (MPT) channel. The loss of membrane potential due to opening of the inner membrane anion channel (IMAC) (Aon et al., 2003, 2007) or the permeability transition pore (PTP) (Zorov et al., 2000) can produce a burst of mitochondrial ROS leading to ROS-induced ROS release (Zorov et al., 2000; Aon et al., 2003; Zhang et al., 2007).

### Major sites of O^.−^_2_generation

- Complex I or NADH: ubiquinone oxidoreductase is considered one of the major source of superoxide production in the ETC complexes (Brand et al., 2004). Electrons from succinate can flow in reverse to complex I to reduce NAD^+^ to NADH. This reverse electron transfer (RET) increases ROS production at the level of complex I. When treated with ADP or malonate, both of which block RET, mitochondrial ROS emission decreases (Liu et al., 2002). Which component of complex I—FMN, Fe-S clusters, or ubiquinone—is producing superoxide is not clear (Brand et al., 2004). Ang II has been shown to damage complex I, thus contributing to ROS generation. Specifically, Ang II is able to down-regulate ND5, a component of complex I, via oxidation of mtDNA thus decreasing electron flow and the likelihood of ROS generation (Ricci et al., 2005).
- Cytochrome *b_c1_* complex or complex III is another important source of O^·−^_2_generation. Coenzyme Q, or ubiquinol (QH2), is the electron donor for complex III. Reduced coenzyme Q (QH2) diffuses to the inner side of mitochondrial membrane to the Q_o_ site, where it transfers one electron to cytochrome c bound to complex III. Cytochrome c then transfers electrons to complex IV. Another QH2 transfers an electron to the oxidized coenzyme Q (Q), reducing it to QH2 near the matrix side of the membrane (Qi site) (Quinlan et al., 2011). After losing an electron to cytochrome c and prior to transferring an electron to Q, ubiquinol may form the intermediate ubisemiquinone (Q.). Electrons can leak (Turrens, 2003) from Q· to oxygen, forming O^·−^_2_. Antimycin, a Qi site inhibitor, elicits large amounts of O^·−^_2_ when O_2_ reacts with ubisemiquinone bound to the Q_o_ site (Murphy, 2009). Under normal conditions, O^·−^_2_generation from complex I and III varies according to tissue conditions…. For example, complex III appears to be the primary source of O^·−^_2_ in heart and lung mitochondria whereas complex I is the major source in the brain (Turrens and Boveris, 1980; Turrens et al., 1982; Barja and Herrero, 1998; Turrens, 2003). In general, acute and chronic infusion of Ang II has been shown to decrease expression of electron transport chain proteins (Larkin et al., 2004). Excessive ROS production due to Ang II can impair complex I and III activities, increasing electron leakage (Prathapan et al., 2014). More information is needed to delineate the role of RAS in the modulation of complex III.
- NADPH oxidase (NOX) family proteins: NOX is a family of transmembrane proteins that may be source of ROS. NADPH oxidase works as a nonspecific host defense system by releasing large amounts of ROS during infections (Thrasher et al., 1994). When cytoplasmic NADPH oxidase is activated, it moves toward membrane-associated cytochrome b558 to form a complex. Cytochrome b558 regulates the enzymatic activity of NADPH oxidase by transferring one electron to molecular oxygen, which gets reduced to O^·−^_2_ (Bayraktutan et al., 1998). While neutrophil NADPH oxidase produces ROS in bursts, vascular NADPH oxidases produce low levels of O^·−^_2_ continuously (Cai et al., 2003). NADPH-derived cytoplasmic ROS can mediate mtKATP opening enabling K+ influx that produces membrane depolarization and alkalinization of the matrix (Di et al., 2007). This matrix alkalization has been shown to increase H_2_O_2_ in the presence of an mtKATP opener (Pain et al., 2000; Heinzel et al., 2005; Andrukhiv et al., 2006; Daiber, 2010).

Location and expression level of the different NOX enzymes determine their function. NOX1 is abundant in colon epithelium and has been reported to play a role in host defense in intestinal crypts and on luminal surface (Szanto et al., 2005). NOX2 is expressed in granulocytes and monocyte/macrophages. ROS generation through NOX2 activation has been shown to play a role in killing microbes and inactivating microbial virulent factors. The exact localization and level of expression of the different NOX enzymes are still not completely sorted out yet. NOX3 has been generally located in the cochlear and vestibular sensory epithelia, and spiral ganglion. NOX4 has been shown to be expressed in the kidney and suggested that it plays a role in protection of the vasculature during inflammatory stress. ROS generation through NOX4 has also been shown to induce HIF-1α transcription factors, which in turn leads to activation of erythropoietin gene in the kidney (Bedard and Krause, 2007; Jung et al., 2008; Schroder et al., 2012). NOX5 is expressed in various tissues including spleen, lymph nodes, and vascular smooth muscle cells (Bedard and Krause, 2007). NOX5 increases Jak2 phosphorylation, which increases vascular smooth muscle cell (VSMC) proliferation in human coronary arteries, aorta, and splenic vessels (Fulton, 2009).

NADPH oxidase is a major source of angiotensin II-induced ROS generation. NOX1 appears to have various roles in VSMCs, including Ang II-induced hypertrophy, serum-induced proliferation, and basic fibroblast-induced growth factor migration (Redmond and Cahill, 2012). It localizes in VSMCs from large arteries (Nguyen Dinh et al., 2013). NOX1 is needed under various physiologic conditions such as thrombin-induced migration, endothelial cell proliferation, cell growth, and vessel formation (Sunggip et al., 2013). Overproduction of O^·−^_2_ from NOX1 and NOX 2 can cause renin release, smooth muscle cell proliferation, and decreased NO^·^, all of which lead to endothelial dysfunction and increased sympathetic tone that in turn cause hypertension (Takac et al., 2012). Ang II can signal via phospholipase D and this pathway may cause VSMC proliferation and contractility through NADPH oxidase (Touyz and Berry, 2002). NOX1distributes in the plasma membrane, primarily generating O^·−^_2_ anions (Dikalova et al., 2005; Valente et al., 2012). Ang II activates NOX1 via NOX1-AT_1_R interaction. This is supported by a study showing that continuous infusion of Ang II was correlated with increased NOX1 mRNA expression (Valente et al., 2012). NOX1 functions are mostly growth-promoting being highly expressed in proliferating cells. It activates VEGF and matrix metalloproteinase, which promote angiogenesis (Lassegue et al., 2001; Wilkinson-Berka et al., 2014). NOX1 is also responsible for O^·−^_2_generation and is implicated in many pathological conditions including atherosclerosis, diabetes and hypertension (Chose et al., 2008; Briones et al., 2011).

NOX4 is found in subcellular compartments such as the nucleus, endoplasmic reticulum, and mitochondria (Wingler et al., 2001; Briones et al., 2011). Unlike NOX1, NOX4 typically generates H_2_O_2_ (Briones et al., 2011; Valente et al., 2012). NOX4 serves as an oxygen sensor and regulates erythropoietin synthesis in the kidneys (Geiszt et al., 2000). In VSMCs, NOX4 is found in focal adhesions and shown to maintain differentiation of cells whereas NOX 1 induces growth and proliferation. This phenomenon was seen in multiple tissues. For example, in coronary arteries, NOX4 expression was correlated with α-actin levels in osteoclasts, and over-expressed NOX4 was associated with increased osteoclast markers. NOX4 is important for maintaining physiologic function of many tissues (Clempus et al., 2007). NOX4 is not up- but instead down-regulated when treated with Ang II (Lassegue et al., 2001; Wingler et al., 2001). Both NOX2 and NOX4 are present in the aortic vascular smooth muscle. When infused with Ang II, NOX2 knock-out mice did not have significant hypertrophy. Therefore, NOX2 was deemed essential for Ang II-induced cardiac hypertrophy and NOX 4 did not seem to play a big role in Ang II-induced cardiac pathologies (Byrne et al., 2003). This argument is supported by many research groups that studied Ang II's effects on ROS generation. Interestingly, Ang II doubled the ratio of O^·−^_2_ to H_2_O_2_ ratio in quiescent cells and almost quadrupled it in proliferating cells. In addition, NOX4 is a source of H_2_O_2_in a variety of vascular cells including fibroblasts, endothelium, and smooth muscles. This constant generation of H_2_O_2_ might be essential to maintaining cell functions in angiogenesis during wound healing (Dikalov et al., 2008). Increased NOX4 expression is essential in tissue survival during ischemic stress, tissue remodeling, and regulation of glutathione. Interestingly, NOX4 has been implicated in cardiac hypertrophy, interstitial fibrosis, and apoptosis in cardiac muscle; however, cardiac function was still maintained (Zhang et al., 2010; Brewer et al., 2011; Schroder et al., 2012; Sunggip et al., 2013). Though some studies show NOX4 playing a role in pathophysiology, the great majority shows that the physiological role of this enzyme is more relevant.

### Role of RAS in ROS scavenging

To maintain ROS balance, the production of ROS has to be matched with a ROS scavenging mechanisms (Figure 1). MnSOD in the mitochondrial matrix converts O^·−^_2_ to H_2_O_2_, which can diffuse across membranes. O^·−^_2_dismutation to H_2_O_2_ is important for signaling and avoiding oxidative damage, in case of oxidative stress (Kang and Pervaiz, 2012). H_2_O_2_ is scavenged by glutathione peroxidase (GPX) and peroxiredoxin (PRX). NADPH generated from mitochondrial transhydrogenase (Hoek and Rydstrom, 1988; Jackson, 2003) is the main electron of the GSH and Trx systems (Nickel et al., 2014). Under normal function, GSH and Trx mitochondrial systems are mainly responsible for offsetting most of the ROS produced by the respiratory chain, especially under state 3 respiration when electron flow is maximal (Aon et al., 2012). Other antioxidants localized to cytoplasmic and intermembrane space include Copper-Zinc superoxide dismutase (Cu-ZnSOD) and catalase (Aon et al., 2012). ROS can mediate increased expression of antioxidant enzymes via activation of the Nrf2-antioxidant response element signaling pathway (Nguyen et al., 2009). ROS-activated Nrf2 increases ARE-mediated gene expression, which includes many antioxidants such as glutathione S-transferase, quinine reductase, and heme oxygenase 1 (Bergelson et al., 1994; Alam et al., 1999). Levels of antioxidants are often decreased in disease states or under oxidative stress. For example, diabetes and insulin resistance are correlated with decreased antioxidant capacity due to decreased SOD and glutathione reductase activities (Aon et al., 2012). Under normal conditions, however, antioxidant enzymes play a major role in scavenging ROS generated from various sources like NADPH oxidase, NOS, xanthine oxidase, and mitochondrial electron transport chain enzymes.

Angiotensin II has been implicated in decreasing the activity of scavenging enzymes, thereby leading to detrimental levels of ROS. Ethanol ingestion is associated with oxidative stress and decreased GSH levels via activation of the RAS. When ethanol-fed rats were treated with the AT_1_R blocker losartan, GSH levels were maintained (Bechara et al., 2005). SOD are also targets of Ang II. Mammals possess three SOD isoforms, Cu-Zn SOD (SOD1), Mn SOD (SOD2), and extracellular SOD (SOD3) located in the cytoplasm, mitochondria and the extracellular space, respectively (Rodriguez-Iturbe et al., 2007). SOD1 is a major defense system against Ang II-triggered ROS in the kidneys. SOD1 knockout mice showed 4-fold increase in afferent arteriolar O^·−^_2_ levels when treated with Ang II (Carlstrom et al., 2010). In addition, in coronary artery disease patients, SOD3 levels were severely reduced in the human arterial walls and this decrease was associated with oxidative stress and reduced NO^·^ bioavailability; these conditions were shown to contribute to the pathophysiology of the disease (Landmesser et al., 2007). When treated with the AT_1_R blocker losartan, an increase in NO^·^ bioavailability and SOD3 activity were seen. A more than 200% increase in SOD3 activity was associated with reduced oxidative stress and improved endothelial function (Hornig et al., 2001). Catalase activity was diminished in Ang II-stimulated cardiomyocytes whereas ROS-elicited by Ang II in mesangial cells implied reduced catalase transcription (Venkatesan et al., 2007; Murtaza et al., 2008; Tan et al., 2008). Reduced catalase mRNA expression and protein levels were noted with Ang II treatment of VSMCs (Xiong et al., 2010). There is also some evidence about the modulation of the Trx system by the RAS system; ACE inhibitors have been shown to improve myocarditis via a mechanism involving the Trx system (Tanito et al., 2004; Touyz, 2004). The RAS not only contributes directly to ROS generation, but also it affects indirectly the redox balance via modulation of various antioxidant enzymes. Treatment methods involving inhibition of RAS enzymes, specifically Ang II type 1 receptor blocker, may be crucial in impeding pathophysiological behavior.

## ROS as signaling molecules

Angiotensin II interacts with various tissues including vascular, renal, and neuronal to induce numerous physiologic signaling cascades and functions. Griendling et al. discussed the role of Ang II in modulating growth-related signaling pathways via ROS signaling (Griendling and Ushio-Fukai, 2000). ROS-mediated oxidation can alter gene expression thorough signaling cascades induction, or interaction with transcription factors. H_2_O_2_ can reversibly inhibit tyrosine phosphatase PTP1B, a regulator of the insulin signaling pathway (Apel and Hirt, 2004; Combs, 2010). Recent research focuses on PTP1B inhibitors as potential therapeutic treatment for type 2 diabetes and obesity (Thareja et al., 2012). ROS also play a role in tyrosine phosphorylation as shown in platelet-derived growth factor where a transient increase in ROS inactivated tyrosine phosphatase (Finkel, 1998). Nontoxic amounts of H_2_O_2_ administered extracellularly have been shown to stimulate mitogen activated protein kinase (MAPK), that directly affects the inflammatory response, cell proliferation, differentiation, and survival in response to extracellular stimuli (Stevenson et al., 1994; Arbabi and Maier, 2002). Exogenous H_2_O_2_ has also been shown to activate JNK, (Finkel, 1998) p38, (Hensley et al., 2000) and NF-_K_B, a protein complex that induces expression of protective genes during inflammation and infection (Baeuerle and Henkel, 1994).

ROS participate in the host innate immune response. Immune cells undergo respiratory burst, producing high levels of free oxygen radicals during infection, a response that results toxic to pathogens (Spooner and Yilmaz, 2011). When a pathogen invades a cell, Leucine-Rich Repeat-containing family member receptor, NLRX1, moves to the mitochondria to stimulate the electron transport chain and initiate ROS production (Arnoult et al., 2009). Since ROS are produced by NADPH oxidases in mature phagocytic cells, many bacteria, like *Francisellatularensis*, interfere with NADPH oxidase assembly (Cirillo et al., 2009). Oxidative burst within macrophages has been shown to decrease urogenital infections associated with pathogen *Chlamydia trachomatis* (Boncompain et al., 2010).

In addition to fighting infections, ROS have also been implicated in cancer therapy since increased ROS levels may be lethal for tumor cells triggering apoptosis. Direct exposure of cancer cells to ROS generating agents like arsenic trioxide, or alternatively inhibiting antioxidant enzymes with2-methoxyestradiol, a SOD inhibitor, may trigger apoptosis in human leukemia (Zhou et al., 2003; Pelicano et al., 2004).

Overall, available evidence indicates that controlled levels of ROS represent effective signaling molecules, intervening in different cellular processes as diverse as communication, inflammation, immunity, and as therapeutic cancer agents. The role of RAS induced ROS in signaling pathways is discussed next.

### Epidermal growth factor (EGF) and extracellular signal-regulated kinase 1/2 (ERK1/2)

EGF is required for Ang II effects on various tyrosine kinases, and its activation is initiated by NOX-induced ROS (Eguchi et al., 1998; Griendling and Ushio-Fukai, 2000; Ushio-Fukai et al., 2001). C-Src, a tyrosine kinase activated by Ang II, promotes multiple signaling events including various MAPKs. Ang II-induced activation of c-Src is redox-sensitive since its stimulation is inhibited by antioxidants (Abe et al., 1997; Ushio-Fukai et al., 2001). EGF receptor activation is needed for Ang II action on ERK1/2, a type of MAPK (Eguchi et al., 1996; Liao et al., 1997; Griendling and Ushio-Fukai, 2000). ERK1/2 has an important role in cell adhesion, cell cycle progression, migration, survival, differentiation, metabolism, and proliferation (Roskoski, 2012). Activation of ERK1/2 is said to be redox-sensitive or redox-insensitive in some studies (Sundaresan et al., 1995; Viedt et al., 2000).

### MAPK and adenilate kinase (Akt)

MAPKs control various cellular activities like growth, apoptosis, and stress signals. Four main MAPKs are ERK1/2, c-Jun N terminal kinase (JNK), p38MAPKs, and big MAPK-1. Ang II can activate JNK, ERK1/2, and p38MAPK. Though there is an ambiguity as to whether ERK 1/2 is redox-sensitive or not, p38MAPK and JNK have been shown to be redox-sensitive when activated by Ang II. Ang II triggers ROS generation through NADPH oxidase activation, followed by JNK and p39MAPK stimulation (Griendling and Ushio-Fukai, 2000; Viedt et al., 2000). In VSMCs, Ang II activates Akt, a serine threonine kinase that plays a role in cell survival and protein synthesis. NADPH-derived ROS induces Akt which is associated with heat shock protein 27, also activated by H_2_O_2_ (Konishi et al., 1997; Coffer et al., 1998; Ushio-Fukai et al., 1999; Griendling and Ushio-Fukai, 2000).

### Nitric oxide synthase (NOS)

NOS is another enzyme capable of producing O^·−^_2_ using tetrahydrobiopterin as a cofactor. Superoxide generated from NADPH oxidases can give rise to peroxynitrite (ONOO^−^) (Figure 1). ONOO^−^ contributes to oxidation of tetrahydrobiopterin, leading to NOS uncoupling and more O^·−^_2_generation (Landmesser et al., 2003). A mitochondrial variant of NOS (mtNOS) can generate nitric oxide (NO^·^), a reactive nitrogen species (RNS). Mitochondrial respiration can be partially inhibited by NO^·^, through inactivation of cytochrome c oxidase from complex IV that can lead to increased ROS production and a vicious cycle of mitochondrial damage by ROS excess (Kang and Pervaiz, 2012). Excess NO^·^ can react with O^·−^_2_ to form the highly reactive ONOO^−^ which, like O^·−^_2_, is unable to permeate the mitochondrial membrane, can cause oxidative damage, nitration and nitrosation (Squadrito and Pryor, 1995; Kang and Pervaiz, 2012). MnSOD can be nitrated by ONOO^−^, decreasing its activity (Quijano et al., 2001). Ang II-treated endothelial cells released more H_2_O_2_, and exhibited mitochondrial loss of membrane potential, respiration impairment, and decreased GSH and NO^·^ formation (Doughan et al., 2008; Daiber, 2010). The reduction of dihydrobiopterin to tetrahydrobiopterin is catalyzed by dihydrofolate kinase. Tetrahydrobiopterin is needed by eNOS for basal NO^·^ production. Ang II can lead to eNOS uncoupling via H_2_O_2_ production in a NOX-dependent manner. This results in down-regulation of dihydrobiopterin kinase and diminished tetrahydrobiopterin cofactor and nNOS uncoupling. Dysfunctional eNOS results in decreased NO^·^ bioavailability (Cai and Harrison, 2000; Sunggip et al., 2013). Ang II-induced O^·−^_2_ can react with endothelial NO^·^ produced by eNOS, decreasing its concentration even more. Loss of NO^·^ contributes to endothelial dysfunction initiating atherosclerosis (Nickenig and Harrison, 2002). In addition, expression of inflammatory molecules like MCP-1 and VCAM-1 appears to be indirectly regulated by Ang II, accelerating atherosclerosis (Nickenig and Harrison, 2002). Angiotensin II-induced phosphorylation of various kinases activates NOX and increases ROS generation.

### Xanthine oxidase (XO)

XO is a major source of ROS. Xanthine oxidoreductase (XOR) degrades purines to generate uric acid. XOR is transcribed as xanthine dehydrogenase, which is converted to XO after oxidation of cysteine residues or proteolysis (Waud and Rajagopalan, 1976; Amaya et al., 1990; Kelley et al., 2010). XO generates both O^·−^_2_ and H_2_O_2_, the latter in greater amounts under low O_2_ and pH, e.g., inflammation, ischemia. Higher O^·−^_2_ levels can be detected under low xanthine concentrations, and high O_2_ tensions (Kelley et al., 2010). Landmesser et al. showed that Ang II administration elevates XO through ROS from NADPH oxidase. NADPH oxidase inhibition decreased XO and O^·−^_2_ levels suggesting that Ang II stimulation of NOX proteins is needed for XO stimulation. Additionally, XO inhibitors like oxypurinol and tungsten markedly reduced Ang II-induced endothelial O^·−^_2_, in agreement with the idea that Ang II stimulates XO. Furthermore, losartan (AT_1_R blocker) treatment decreased the endothelial levels of both XO and O^·−^_2_. Patients with coronary disease treated with AT_1_R blocker for 4 weeks displayed reduced endothelial XO activation compared to the placebo (Landmesser et al., 2007).

### Other enzymes

Other enzymes such as pyruvate dehydrogenase, α-ketoglutarate dehydrogenase, glycerol-3-phosphate dehydrogenase, and from fatty acid β oxidation can also contribute to ROS generation (Kang and Pervaiz, 2012). Ang II can modulate the activity of these various enzymes. For instance, many studies have shown effects of Ang II on fatty acid oxidation and nonalcoholic fatty liver disease (Kurita et al., 2008; Toblli et al., 2008). Increased Ang II levels cause mitochondrial oxidative damage, which leads to impairment of beta oxidation causing hepatic steatosis. Treatment with AT_1_R blockers like valsartan produced substantial improvement of mitochondrial abnormalities (Monteiro et al., 2005). The mechanism by which fatty acid oxidation appears to be mediated by ROS generated from NADPH oxidase as well as decreased Cu, Zn SOD activity (Wei et al., 2009). Ang II has also been shown to promote pyruvate dehydrogenase complex acetylation, leading to decreased glucose oxidation (Mori et al., 2013). α-ketoglutarate dehydrogenase activity was shown to be increased by Ang II treatment. Increased cytoplasmic and mitochondrial free Ca^+2^ levels are associated with hepatic stimulation via Ang II. Since α-ketoglutarate dehydrogenase function is positively stimulated by Ca^+2^, its activity is indirectly influenced by Ang II (Exton, 1985; Williamson et al., 1985; Rashed et al., 1988).

Together, the evidence available indicates that RAS can induce ROS generation through direct—influencing various signaling pathways, as well as indirect—modulating activities of antioxidant enzymes, mechanisms.

### Aging, mitochondria and RAS associated pathology

Aging is associated with the accumulation of various deleterious changes in cells. According to the free radical and mitochondrial theory of aging, mitochondria initiate most of the deleterious changes in aging and govern life span (Harman, 1956, 2006; Cadenas and Davies, 2000; Cadenas, 2004). Three key mitochondrial functions that become dysregulated with aging are: (1) ROS production, (2) ATP synthesis, and (3) apoptosis (Conley et al., 2007). As proposed by the mitochondrial theory of aging, increased mitochondrial ROS generation precipitates mitochondrial DNA damage and mutations, which in turn leads to failed oxidative phosphorylation and diminished ADP/ATP reservoir (Harman, 1956, 2006), ultimately contributing to mitochondrial deterioration and activation of cell death pathways (Echtay et al., 2002; Dirks et al., 2006; Skulachev, 2006; Conley et al., 2007; Pandur et al., 2014). Moreover, mitochondrial morphodynamics through fusion and fission can also be altered with aging could potentially lead to their dysfunction (Bleazard et al., 1999; Chen et al., 2003; Yu et al., 2012). Chronically activated ROS has been implicated in mitochondrial energetic impairment along with the development and progression of a host of aging-related conditions including atherosclerosis, myocardial hypertrophy, vascular dysfunction, hypertension (Heymes et al., 1998; Wang et al., 2003; Kimura et al., 2005; Doughan et al., 2008; Fukai, 2009; Min et al., 2009; Widder et al., 2009), type 2 diabetes, frailty, heart failure, neurodegeneration (including Alzheimer's disease, Horan et al., 2012), and sarcopenia (Ballinger et al., 1994; Wallace, 2001, 2005, 2010, 2011; Loeb et al., 2005; Conley et al., 2007; Moore et al., 2010). Clinically, the onset of this mitochondrial failure is difficult to estimate, however, the accumulation of damaged mitochondria typically appears in humans by mid to late seventies and once established is thought to be irreversible (Aiken et al., 2002; Herbst et al., 2007). Recently, several studies have shed light on early mitochondrial changes in healthy subjects that predate the accumulation of damaged mitochondria by almost a decade. These studies have demonstrated that increased uncoupling leads to a reduction in mitochondrial efficiency in otherwise healthy people in their sixties (Greco et al., 2003; Hutter et al., 2004; Bua et al., 2006; Mogensen et al., 2006; Amara et al., 2007).

The major mechanism by which dysfunction occurs is via mtDNA mutations. Unlike nuclear genome, mitochondrial genome is circular, and is not condensed around histones or packed tightly. This makes it less protected and more easily damageable than nuclear DNA (nDNA) (Croteau et al., 1999). mtDNA damage is mostly attributed to ROS. AngII can stimulate mitochondrial ROS production via activation of cytoplasmic NOX-derived O^·−^_2_and through direct effects on mitochondria as well. Administration of antioxidants inhibited Ang II effects on AP-1 signaling pathway (Puri et al., 1995; Xia et al., 1998; de Cavanagh et al., 2007). Further evidence in support of Ang II action on mitochondria is given by research showing AT_2_R co-localization with this organelle likely in inner membrane, in various tissues. Ang II and AT_2_R are likely generally present in the inner membrane of mitochondria (Inagami, 2011). Ang II stimulates production of NO^·^ via activation of calcium/calmodulin-dependent eNOS, including mRNA and protein expression levels (Brown, 1999; Yan et al., 2003). mtDNA codes for critical proteins participating in oxidative phosphorylation (Croteau et al., 1999). In addition, mtDNA has less repair mechanisms compared to nDNA; for example, mitochondria lack nucleotide excision repair mechanisms (Larsen et al., 2005). mtDNA mutation rate is shown to increase with age, affecting liver, skeletal muscles, and cardiac muscles (Katayama et al., 1991; Corral-Debrinski et al., 1992; Marin-Garcia et al., 2002; Druzhyna et al., 2008). Excess ROS generation may overwhelm antioxidant enzymes, thereby preventing the mitochondria from protecting themselves (Golden and Melov, 2001). With chronic exposure to elevated ROS and decline in repair mechanisms, mtDNA mutations accumulate during aging (Lin and Beal, 2006). Under RAS over-activation- as seen in diabetes, hypertension and aging- Ang II induced ROS plays a significant role in tissue damage. Ang II induced mtROS generation has been implicated in atherosclerotic lesions and impairment of cardiac respiration and TCA cycle function leading to disease (Pueyo et al., 2000; de Cavanagh et al., 2007).

In cardiovascular tissue, mitochondrial ROS contribute to senescence of endothelial cells and chronic low-grade vascular inflammation (Ungvari et al., 2007). These endothelial cells can contribute to atherosclerosis by suppressing regeneration and angiogenesis of endothelium in the vascular wall leading to cardiovascular aging (Dai et al., 2012) and the development of hypertension (Heymes et al., 2003; Shiomi et al., 2004; Wang et al., 2010; Sugamura and Keaney, 2011; Dikalov and Ungvari, 2013).

Development and progression of several neurodegenerative disorders has also been linked to mitochondrial dysfunction (Beal, 2007). A significant decrease in mitochondrial coupling efficiency in primary hippocampal neurons, reduced steady state basal respiration, and decreased ATP turnover were noted in several neurodegenerative disorders (Horan et al., 2012). In Alzheimer's disease, several studies have demonstrated that oxidative damage appears to increase development of intracellular Aβ plaques. ROS activation of c-Jun N terminal kinase and p38 mitogen activated protein kinase lead to increased activity of β secretase, causing increased Aβ levels (Nishida et al., 2006; Beal, 2007). Parkinson's disease is caused by a recessive mutation of DJ1 that leads to hypersensitivity to MPTP and oxidative stress since DJ1 protects against oxidative stress-induced cell death. Studies show that treatment with SOD1 and vitamin E decrease degeneration of dopaminergic neurons (Bonifati et al., 2003; Kim et al., 2005; Wang et al., 2006a). Huntington's disease (HD) is also associated with oxidative stress since reduced activity of Complex II and III is seen in basal ganglia and cortex. In addition, HD patients show decreased PGC1α, which suppresses ROS by activating ROS scavenging enzymes (St-Pierre et al., 2006; Weydt et al., 2006; Beal, 2007). ROS are implicated not only in neurodegenerative disorders but also in various other diseases like age-related musculoskeletal disorders. In sarcopenia, a disease characterized by reduced skeletal muscle mass with aging, electron transport system abnormalities were seen in muscle fibers with “ragged red” phenotype and these changes were associated with loss of muscle mass (Bua et al., 2002). Patients with Type 2 diabetes show decreased antioxidant capacity and possibly an increase in ROS generation by leukocytes (Mohanty et al., 2000). Studies show increased expression of ROS markers in pancreatic islet cells under diabetic conditions. Furthermore, β cells are much more sensitive to ROS due to decreased antioxidant capacity. Chronic hyperglycemia increases ROS levels while decreasing binding of the transcription factors PDX-1 and Maf-A from pancreas that exacerbates suppression of insulin synthesis and release. In addition, treatment with antioxidants such as N-acetyl-L-cysteine and taurine, diminished insulin resistance due to hyperglycemia (Kaneto et al., 2010).

The overlap between age related changes in RAS and mitochondria and the implications of this overlap on age-related diseases are quite complex. Studies shows that Ang II contributes to plaque rupture by initiation of VSMC apoptosis, which could be prevented by AT_1_R blockers (Lemay et al., 2000). Ang II induces metalloproteinase activity, which is involved in collagen breakdown and matrix degradation, indirectly through ROS (Shah and Galis, 2001; Nickenig and Harrison, 2002). Ang II can induce interleukin-6 (IL6), leukemia inhibitory factor, and cardiotrophin-1 in cardiac fibroblasts (Sano et al., 2001). IL-6 is an inflammatory marker and a high level of which is associated with mortality due to increased progression of cardiovascular disease (Volpato et al., 2001). Leukemia inhibitory factor is a key player in cardiac hypertrophy (Kodama et al., 1997). Cardiotrophin-1 plays a role in heart failure since it can induce ventricular remodeling by activating cardiomyocyte hypertrophy and collagen synthesis (Calabro et al., 2009).

Many studies highlighted the role that RAS plays in hypertension and cardiovascular disease (Marchesi et al., 2008). By activating local mediators, like vascular endothelial growth factor (VEGF) and prostaglandins, such as leukotriene C4, PGE2, and PGI2, Ang II plays a critical role in regulating vascular permeability during hypertension (Harris et al., 2004). Ang II-stimulated release of these local factors in VSMCs leads to angiogenesis, vascular permeability, and inflammation. In particular, studies have shown that AT_1_R activation causes VEGF secretion (Suzuki et al., 2003). Hypertension is a condition that results from ROS of vascular origin produced by elevated levels of Ang II. Chronic administration of Ang II in mice, triggered elevated O^·−^_2_ from mitochondria as compared to controls (Widder et al., 2009). O^·−^_2_ and H_2_O_2_ produced due to elevated Ang II can carry out various actions in VSMCs—phosphorylation of MAP kinases, induction of proto-oncogenes, and activation of AP-1; H_2_O_2_ can induce PDGF stimulation of STATS. All these signaling events contribute to vascular wall remodeling and thickening seen in hypertension (Touyz, 2000). Ang II via AT_1_R also affects brain tissue by increasing neuronal firing rate and activity. In neurons, NADPH oxidase-derived ROS produced due to Ang II signaling increase intracellular Ca^+2^ concentration (Wang et al., 2006b), which in turn can stimulate mitochondrial O^·−^_2_ stimulates mitochondrial superoxide generation (Hongpaisan et al., 2004). In rostral ventrolateral medulla, these effects may influence blood pressure, causing baroreflex abnormalities in chronic hypertension (Nozoe et al., 2008).

RAS has been implicated in many pathological conditions. Ang II can precipitate mild to severe mitochondrial dysfunction in addition to ROS generation. Amelioration of age-related renal mitochondrial dysfunction under hypertensive conditions, and ischemic injury, has been described in patients treated with angiotensin receptor blockers (Doughan et al., 2008). Losartan treatment prevents mitochondrial dysfunction and structural changes in the kidney while up regulating antioxidant enzymes, maintaining GSH and MnSOD levels, and attenuating uncoupling proteins. These effects were not apparent when patients were treated with a Ca^2+^ channel blocker allowing to conclude that Ang II must play a role in mitochondrial dysfunction (de Cavanagh et al., 2006).

Since Ang II is linked to NOX, Wosniak et al. (2009) examined the effects of mild mitochondrial uncoupling and Ang II stimulation of NOX isoform in VSMCs. Ethidium bromide was used to induce mild mitochondrial stress, which the investigators described as “neither rapidly lethal nor promoting profound redox derangements” (Wosniak et al., 2009). Results showed that Ang II-induced NOX activation can be completely eliminated with mild mitochondrial uncoupling. Ang II up regulates NOX 1 expression and down regulates NOX4 expression and with mild mitochondrial dysfunction there was a decrease in NOX1 and increase in NOX4. Therefore, these authors concluded that functional mitochondria are required for Ang II-induced NOX activation. Wosniak et al. also noted that mitochondrial function influences activation of growth factor receptors involved in NOX signaling (Wosniak et al., 2009). In addition, when Ang II was administered, increased mtDNA damage was only observed in those cells that had existing dysfunctional mitochondria. Cells with functional mitochondria did not show any markers of mtDNA damage. The researchers speculated that mitochondria might be acting as a switch between normal to pathological effects of Ang II (Wosniak et al., 2009). The relationship between angiotensin-related mitochondrial ROS and NADPH oxidase is still a novel area of research that can potentially provide insight into pathophysiology of many diseases and may pave the way for new therapeutic approaches.

### The use of angiotensin receptor blockers (ARBs) in mitochondrial dysfunction

Elevated Ang II produces increased levels of ROS (by activating NADPH oxidase and various other enzymes) that contribute to various pathological conditions. AT_1_R blockade decreases RAS-mediated activation of NADPH oxidase and oxidative stress, leading to reduced left ventricular fibrosis and mitochondrial remodeling (Whaley-Connell et al., 2008). Losartan treatment also reverses left ventricular hypertrophy, reduces fibrosis, ultimately causing an overall improvement of cardiac function (Khaper and Singal, 2001). Various studies have shown a lower rate of mortality from cardiovascular disease in patients treated with angiotensin receptor antagonists. Beneficial effects of angiotensin receptor blockers include lowered cerebral lesion incidence, reduced cardiac hypertrophy, and reduced glomerulosclerosis. Eprosartan was shown to be effective in preventing cardiac remodeling, renal failure, and decreasing mortality (Takemori et al., 2005). Interestingly, insulin treatment induces AT_1_R overexpression, and in diabetics, ARBs have been shown to effectively preserve renal function and reduce cardiovascular endpoints (Nickenig et al., 1998; Nickenig, 2004). ARBs reduces Ang-II induced lipolysis and adipocyte dysfunction (Takemori et al., 2013). Contrary to AT_1_R, the effects of AT_2_R are considered protective. ARBs may increase activation of AT_2_ receptor augmenting end-organ protection (Carey et al., 2000; Unger, 2002). More research on ARBs and activation of AT_2_ receptor might yield promising results in treating various pathological conditions.

AngII receptor blockers have several features in common: high affinity for AT_1_R and almost no affinity for AT2 receptors; high protein binding capacity behaving as competitive inhibitors with slow dissociation (Burnier, 2001). There are six common types of ARBs used currently as treatment for hypertension—losartan, valsartan, candesartan cilexetil, irbesartan, eprosartan, and telmisartan. These ARBs have been shown to protect against various disease states. For example, in rats with myocardial infarction, losartan improves function of several antioxidant enzymes, significantly reducing oxidative stress. In particular, losartan increases the activity of GSH peroxidase (Khaper and Singal, 2001) while decreasing levels of vascular O^·−^_2_ due to its antagonist activity on AT_1_ receptors (Kurz et al., 1999). It also restores NO^·^ synthesis and availability playing its role as an antihypertensive drug protecting endothelial cells (Qadri et al., 2001). Valsartan is effective in improving heart mitochondrial function under acute ischemia (Monteiro et al., 2005). Long-term treatment of rabbit heart with valsartan, after infarction, showed reduced lipid peroxide levels; an improvement in postinfarct ventricular remodeling and coronary endothelial dysfunction was also seen (Kuno et al., 2002). In diabetic patients, candesartan cilexetil has been shown to improve pancreatic β cell function (Qadri et al., 2001) and irbesartan was effective against postprandial hyperglycemia and hypertriglyceridemia on endothelial function (Ceriello et al., 2005). Telmisartan can inhibit apoptosis, oxidative stress, and neuro-inflammation in addition to its antihypertensive functions (Beckman et al., 1990; Butler et al., 2003; Monteiro et al., 2005; Shao et al., 2006). In hypertensive patients, ARBs were shown to improve normal retinal perfusion and endothelium-dependent vasodilation in coronary and renal circulation (Delles et al., 2004). In addition, ARBs have been associated with a reduction in inflammatory markers. This is of particular importance since low grade chronic inflammation is associated with many neurodegenerative diseases like Alzheimer's and Parkinson's diseases, amyotrophic lateral sclerosis, multiple sclerosis, Huntington's disease and frailty (Tracy, 2003; Gao and Hong, 2008; Holmes et al., 2009). ARBs are also able to reverse early myocardial impairment (Cadeddu et al., 2010), improve insulin sensitivity, decrease incidence of type 2 diabetes (Saitoh et al., 2009) and prevent renal fibrosis (Shao et al., 2006).

## Prospective

Mitochondrial dysfunction and oxidative stress underlie many pathologies and constitute primary theories of aging. Understanding how age-related mitochondrial dysfunction might be mitigated or exacerbated is critical to advancing this research field. The RAS is currently regarded as a physiological system of vital importance because of its links to both mitochondrial function/dysfunction and a host of age-related diseases. Although many prior studies have advanced our understanding in each of these aging-relevant biological systems, the progress in delineating the molecular mechanisms involved has been rather slow. Given the availability of selective, and relatively safe blockers of RAS, studies focusing on the interface between mitochondria, RAS, and aging may prove to be very important in clinical translation of research.

### Conflict of interest statement

The authors declare that the research was conducted in the absence of any commercial or financial relationships that could be construed as a potential conflict of interest.
